# Risk factors for *Mycobacterium ulcerans* infection (Buruli Ulcer) in Togo ─ a case-control study in Zio and Yoto districts of the maritime region

**DOI:** 10.1186/s12879-018-2958-3

**Published:** 2018-01-19

**Authors:** Issaka Maman, Tchadjobo Tchacondo, Abiba Banla Kere, Ebekalisai Piten, Marcus Beissner, Yiragnima Kobara, Komlan Kossi, Kossi Badziklou, Franz Xaver Wiedemann, Komi Amekuse, Gisela Bretzel, Damintoti Simplice Karou

**Affiliations:** 1 Institut National d’Hygiène (INH), National Reference Laboratory for Buruli ulcer disease in Togo, 26 QAD Rue Nangbeto, 1BP, 1396 Lomé, Togo; 2 Ecole Supérieure des Techniques Biologiques et Alimentaires (ESTBA), Laboratoire des Sciences Biologiques et des Substances Bioactives, Université de Lomé, Lomé, Togo; 3 Centre National de Référence pour le Traitement de l’Ulcère de Buruli (CNRT-UB), Centre Hospitalier Régional (CHR) de Tsévié, Lomé, Togo; 4 Department for Infectious Diseases and Tropical Medicine (DITM), Medical Center of the University of Munich (LMU), Munich, Germany; 5 Programme National de Lutte Contre l’Ulcère de Buruli, la Lèpre et le Pian (PNLUB-LP), Lomé, Togo; 6 German Leprosy and Tuberculosis Relief Association (DAHW-T), Togo office, Lomé, Togo

**Keywords:** Buruli ulcer, *Mycobacterium ulcerans*, District of Zio, District of Yoto, Togo, Risk factor, Case-control study

## Abstract

**Background:**

Buruli ulcer (BU) is a neglected mycobacterial skin infection caused by *Mycobacterium ulcerans*. This disease mostly affects poor rural populations, especially in areas with low hygiene standards and sanitation coverage. The objective of this study was to identify these risk factors in the districts of Zio and Yoto of the Maritime Region in Togo.

**Methods:**

We conducted a case-control study in Zio and Yoto, two districts proved BU endemic from November 2014 to May 2015. BU cases were diagnosed according to the WHO clinical case definition at the Centre Hospitalier Régional de Tsévié (CHR Tsévié) and confirmed by Ziehl-Neelsen (ZN) microscopy and IS2404 polymerase chain reaction (PCR). For each case, up to two controls matched by sex and place of residence were recruited. Socio-demographic, environmental or behavioral data were collected and conditional logistic regression analysis was used to identify and compare risk factors between BU cases and controls.

**Results:**

A total of 83 cases and 128 controls were enrolled. The median age was 15 years (range 3–65 years). Multivariate conditional logistic regression analysis after adjustment for potential confounders identified age (< 10 years (OR =11.48, 95% CI = 3.72–35.43) and 10–14 years (OR = 3.63, 95% CI = 1.22–10.83)), receiving insect bites near a river (OR = 7.8, 95% CI = 1.48–41.21) and bathing with water from open borehole (OR = 5.77, (1.11–29.27)) as independent predictors of acquiring BU infection.

**Conclusions:**

This study identified age, bathing with water from open borehole and receiving insect bites near a river as potential risk of acquiring BU infection in Zio and Yoto districts of the Maritime Region in south Togo.

**Electronic supplementary material:**

The online version of this article (doi: 10.1186/s12879-018-2958-3) contains supplementary material, which is available to authorized users.

## Background

Buruli ulcer (BU) is an emerging skin disease caused by an infection with *Mycobacterium ulcerans* [1–4]*.* BU represents the third most common mycobacterial disease after tuberculosis and leprosy in immunocompetent hosts. Infection with *M. ulcerans* often leads to extensive destruction of skin and soft tissue with the formation of large ulcers, commonly on limbs. About 60% of lesions occur on the lower limbs, 30% on the upper limbs and 10% on the rest of the body. Although the rate of mortality of Buruli ulcer is low, the serious morbidity caused by the disease includes functional disabilities that may result in permanent social, economic and developmental problems. At least 50% of those affected by BU are children aged < 15 years. Rate of infections among males and females are equal [1–5]. To date, BU cases have been reported in over 30 countries, particularly in tropical and subtropical climate regions but also in temperate climate zones such as Japan and southern Australia [1–5]. BU is a neglected tropical disease (NTD) with a poorly known global prevalence and mainly affects remote rural African communities [6]. According to the WHO, from an estimated 7000 BU cases reported annually (2016) worldwide and more than 4000 cases occurred in Sub-Saharan Africa. The largest numbers of reported BU cases were from West African countries, particularly from Ivory Coast (about 2000 cases annually), Benin and Ghana as well, each of which reported about 1000 cases a year (2016) [1–6].

In Togo, the first cases of BU have been described in 1996 by Portaels et al. [7]. From 1996 to 2004, more than 100 cases were clinically diagnosed [8, 9]. Between 2007 through 2010 [9], a joint research project between the German Leprosy and Tuberculosis Relief Organization in Togo (DAHWT) and the Department for Infectious Diseases and Tropical Medicine, University Hospital, Ludwig Maximilians-University, Munich, (Germany) allowed the first systematic study of laboratory confirmed BU cases from Togo and established prevalence of BU in the Maritime Region of south Togo. Since 2011, within the frame of the European Community funded research project “BuruliVac”, a National Reference Laboratory for BU (NRL-UB) was established at the Institut National d’Hygiène (INH) and all BU cases notified were confirmed by IS*2404* PCR [10].

Previous case-control studies [11–15] have reported a high risk of contracting Buruli ulcer by swimming in or wading through a river. Residence near marshy areas with stagnant or slow-flowing water bodies and farming activities near rivers were additionally described as risk factors [11–15]. Several epidemiologic studies in Africa [16–19] and Australia [20, 21] have identified aquatic sources as possible reservoirs of *M. ulcerans* by detecting DNA of the pathogen in water filtrant and in a range of environmental samples. All these findings used PCR methodology which does not provide definitive proof for the presence of intact bacteria in a matrix. More recently, results from laboratory experiments [22–25] have suggested a new hypothesis that aquatic insects, fish, plants and terrestrial mammals may be reservoirs for *M. ulcerans* and that insect may be even involved in transmission to humans. In addition, the successful culture of *M. ulcerans* from an aquatic water bug collected in Benin [26] provides definitive evidence for the presence of *M. ulcerans* in an aquatic invertebrate as possible reservoirs or vectors of *M. ulcerans*.This considerable achievement showed that the *M. ulcerans* is present in the environment and that transmission to humans might occur through contact with water or environmental samples contaminated with or harboring the mycobacteria [27]. Inoculation of this pathogen into the subcutaneous tissue could occur when the exposed skin is traumatized. However, the exact mechanism of transmission of the bacterium remains unclear [27].

Human-linked changes in the aquatic environment such as dam constructions on rivers, deforestation, agriculture and mining have led to environmental disturbance and may contribute to the spread of *M. ulcerans* [28, 29]*.* This could increase the incidence of Buruli ulcer cases in endemic areas and lead to the emergence of *M. ulcerans* in areas where the pathogen was previously absent [28]. Some studies, mainly clinical [7–10, 30–32], were carried out in Togo on BU but little were focused on socio-demographic, environmental or behavioral factors. We conducted this study to determine such risk factors for *M. ulcerans* infection in the Zio and Yoto Districts in the Maritime Region.

## Methods

### Study design

**Fig. 1:**
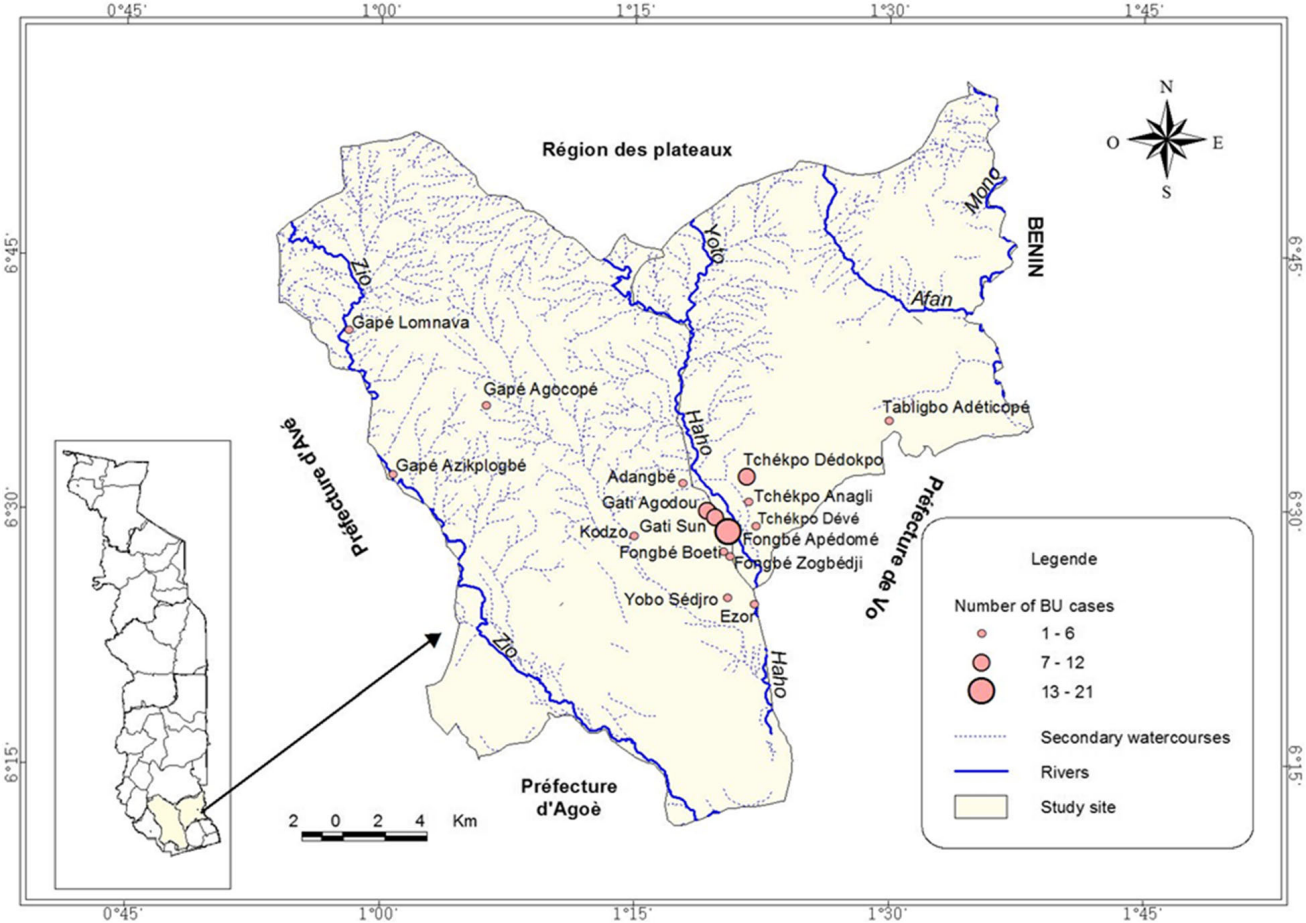
Maritime Region Map presenting villages surveyed, distribution of BU cases and hydrographic network: The circles in red correspond to the number of Buruli ulcer cases and placed at the 17 villages location in Districts of Zio and Yoto of the Maritime Region. Most of BU cases are located arround the watercourse of Haho with few cases observed near the Zio watercourses. These watercourses are main sources of activities with water contact that are associated with increasing risk of *M. ulcerans* infection

We conducted a case-control study in the Zio and Yoto districts of the maritime region (Fig. 1) between November 2014 and May 2015. Buruli ulcer cases were selected at the National Reference Center for BU Treatment (CNRT-UB) located at CHR Tsévié. Patients enrolled were recruited from March 2013 to May 2015. Controls were recruited by active search during the survey. Patients infected with the human immunodeficiency virus (HIV) or with active tuberculosis were excluded from the study.

### Case definition

A probable case of Buruli ulcer was defined as any person aged ≥2 years who lived in Zio or Yoto district showing clinical symptoms according to the WHO clinical definition of BU [3]. A confirmed case was defined as a probable case with detection of *M*. *ulcerans* using Ziehl-Neelsen (ZN) microscopy and IS*2404* PCR [9, 10].

### Recruitment of controls

An eligible control was defined as any person aged ≥2 years without any history or clinical symptoms of Buruli ulcer. Up to two controls were randomly selected and matched to cases by sex and place of residence (home where lived the case or neighbor home in the same village).

### Study site

This study was conducted in 17 villages in districts of Zio and Yoto where more than 85% of confirmed BU patients originate. These districts are in the Maritime Region (South of Togo) which covers an area of ​​6.359 km^2^. With an estimated population of 1,762,518 inhabitants in 2012, the climate is tropical and humid with two rainy seasons and two dry seasons. The maritime region has a flat topography, with a low contrast characterized by a sedimentary basin that covers 4/5 of the region, a low altitude (50-80 m on average) and crossed by the depression of the Lama. The clay soil remains soggy and muddy in the rainy season. Water stagnates for several months in this region. The hydrographic network comprises 3 large rivers which are the Mono in the east, the Zio and the Haho in the center with several small tributaries that flow into the “lac Togo” (Fig. 1). All these streams have a low flow, closely linked to seasonal variations of precipitations [33].

### Laboratory confirmation

Sample collection: Samples were collected according to standardized procedures as previously described [9, 10]. Briefly, fine needle aspirates (FNA) were collected from the center of non-ulcerative lesions or from undermined edges of ulcerative lesions including necrotic tissue. Swabs were collected by circling the entire undermined edges of ulcerative lesions. Samples taken were put in tubes containing cell lysis solution (CLS, Qiagen, Hilden, Germany) and sent for PCR analysis at INH.

Laboratory testing: Direct smears for microscopy were prepared from swab and FNA samples at peripheral care units or CNRT-UB and subjected to Ziehl-Neelsen staining for detection of acid fast bacilli at the laboratory of the CHR. Slides were analyzed by microscopy according to the WHO [34] recommended grading system. All slides were double checked at the INH by a second technician for external quality control.

All molecular analyses were conducted at the NRL for BU at INH as previously described [10]. For PCR analysis, DNA was extracted from FNA and swab samples with the Gentra Puregene DNA extraction kit (Qiagen) with minor modifications of the manufacturer’s protocol. The conventional IS*2404*-PCR with gel-based amplicon detection was applied using dry-reagent-based consumables (DRB-IS*2404* PCR). Briefly, for the DRB-PCR, the primers MU5 (AGCGACCCCAGTGGATTGGT) and MU6 (CGGTGATCAAGCGTTCACGA) were lyophilized in reaction tubes. The Illustra PuReTaq Ready-To-Go PCR beads (GE Healthcare) containing Taq polymerase, dNTPs and Mg^2+^ were added and dissolved in water before adding DNA template. All PCR assays included negative extraction, positive, negative (no template) and inhibition controls. All inhibited samples were repeated after 10-fold dilution.

### Sample size

We used the power calculation tool of Epi-Info (version 7; 2012) to determine the sample size by setting α to 5% and power β to 80%. The health and population development survey (EDST; 2014) in Togo showed that 36,2% of households use water from unprotected sources [35, 36]. The odds ratio (OR) of the association between cases and controls was set at 2, yielding a sample size of 100 BU cases and 200 controls.

### Data collection

The survey was conducted by a team of four people including a clinician from CHR Tsévié, a focal point of the national program of BU surveillance, a community volunteer and a laboratory technician. Case residence was identified by the community volunteer. Once at home, we selected one or two matched control subjects. A well-structured questionnaire was administered to all selected participants (Additional file 1: Questionnaire form S1). For participants who could not respond in French, the interview was conducted in the local language. For children under 10 years, we interviewed their parents mainly for their activities and behavior. All the participants gave their consent prior to data collection on socio-demographic characteristics, behavior, occupational and environmental factors as well as administration of BCG vaccination.

### Statistical analysis

Data collected were entered in a database designed through Epi-Info software (Version 7; 2012). Statistical analysis was carried out by SPSS software (Statistical Package for Social Science, Version 16.0, SPSS Inc. and Chicago, IL). Qualitative data were presented as number n (%) and quantitative data as mean ± standard deviation. Buruli ulcer was considered as the dependent variable and socio-demographic characteristics, occupational and environmental factors as independent variables. Student t-test was used for comparison of mean or median age and number of people in the household between patients and controls with significant level set at *p* ≤ 0.05. Univariate logistic regression was used to determine the risk factors of *M. ulcerans* infection by determining the odds ratio (OR) and 95% confidence interval (CI). All variables obtained from the univariate analysis with *p*-value ≤0.1 were retained for the multivariate model. The final model was obtained after a step-by-step backward elimination step using multiple logistic regressions.

## Results

Clinical diagnosis, laboratory confirmation and characteristics of BU cases.

During the study period, 129 probable cases were observed (Table 1). ZN microscopy confirmed the presence of acid-fast bacilli (AFB) in 67 (52%) among probable cases while PCR detected *M. ulcerans* DNA in 91 cases (71%). The two techniques were both positive in 58 cases (44.5%) and no AFB were detected from any of the PCR negative lesions (Table 1). Of all confirmed cases, lesions were mainly ulcers (41.7%), nodules (27.5%) and plaques (19.8%) (Table 2). Most of these lesions were found on the lower (40%) and the upper limbs (45%). The rest of lesions were localized on the buttocks, abdomen, back and head. Of 91confirmed cases, 83 (91%) responded to the questionnaire. The remaining 8 cases were absent at the time of the survey. Therefore, the case-control study was carried out with 83 cases and 128 control subjects. The socio-demographic characteristics of the participants are presented in Table 3.

**Table 1:** Yearly distribution of clinically suspected BU cases, laboratory tests used for confirmation and positive BU cases detected in Zio and Yoto Districts of Maritime Region, Togo, March 2013 to April 2015

Parameters	Number of BU suspected cases, n
Yearly distribution of BU suspected cases	
2013 (March to December)	31
2014	63
2015 (January to April)	35
Total	129
Laboratory confirmation tests	Positive BU Cases, n (%)
Ziehl-Neelsen microscopy (129 cases analyzed)	67 (51.9)
PCR technique (129 cases analyzed)	91 (70.5)
ZN microscopy and IS2404 PCR	58 (44.4)

**Table 2:** Type and localization of observed lesions in 91 BU cases in Zio and Yoto Districts of Maritime Region, Togo, March 2013–May 2015

Clinical Characteristics	Number of BU cases, n (%)
Type of lesions	
Edema	10 (10.9)
Nodule	25 (27.5)
Plaque	18 (19.8)
Ulcer	38 (41.7)
**Total**	**91(100.0)**
Localization of lesions	
Abdomen	3 (3.0)
Back	2 (2.5)
Buttocks	3 (3.0)
Head	2 (2.5)
Lower limbs	37 (40.6)
Upper limbs	44 (48.3)
**Total**	**91 (100.0)**

**Table 3:** Socio-demographic characteristics of the participants of the case-control study in Zio and Yoto Districts of the Maritime Region, Togo, May 19–30, 2015

Characteristics	Cases n (%)	Controls n (%)	Total n (%)	p*
Number of participants	83 (39.3)	128 (60.7)	211	
Sex				0.32
Female	50 (60.2)	68 (53.1)	118 (55.9)	
Male	33 (39.8)	60 (46.9)	93 (44.1)	
Age				
Median (range in years)	11 (3–65)	19 (8–60)	15 (3–65)	**0.001**
< 10	39 (47.0)	15 (11.7)	54 (25.6)	0.01
11–14	16 (19.3)	19 (14.8)	35 (16.6)	0.03
15–24	13 (15.7)	47 (36.7)	60 (28.4)	0.76
> = 25	15 (18.1)	47 (36.7)	62 (29.4)	
District of residence				0.75
Zio	62 (74.7)	98 (76.6)	160 (75.8)	
Yoto	21 (25.3)	30 (23.4)	51 (24.2)	
Education level				**0.03**
None	19 (22.9)	36 (28.1)	55 (26.1)	
Primary school	57 (68.7)	67 (52.3)	124 (58.8)	
Secondary school	7 (8.4)	25 (19.5)	32 (15.2)	
Ethnicity				0.48
Ewe	76 (96.2)	110 (97.3)	186 (96.9)	
Other (Lamba. Moba et Peulh)	3 (3.8)	3 (2.7)	6 (3.1)	
Number of people in household				0.58
Median (range)	8.5 (2–40)	8.0 (3–40)	8.0 (2–40)	

*Boldface type indicates differences that were statistically significant at *p* < 0.05 between cases and controls

### Univariate analysis

#### Socio-demographic characteristics of the participants

Most of BU patients (66%) were under 15 years of age and were significantly younger (median age = 11 years) compared to controls (median age = 19 years with 73% of who aged more than 15 years) (*p* = 0.001) (Table 3). The primary school educational level was more frequent (59%) (*p* = 0.03) in children aged ≤10 years (28.2%) while the secondary school educational level was associated with the 15–24 age groups (56.2%) (*p* = 0.007) (Table 3). Among cases, women (60%) were more frequently affected than men (40%) (*p* = 0.01). There was no significant difference in the number of people living per household between cases and controls (*p* = 0.58) (Table 3).

### Environmental factors

#### Exposure to water contact

We found that drinking or washing clothes with water taken from rivers (*p* = 0.95), open boreholes (*p* = 0.98) and boreholes with pump (*p* = 0.49) were not associated with an increased risk of contracting Buruli ulcer (Table 4). However, bathing with water from an open borehole was associated with higher risk of contracting BU (OR = 5.07, 95% CI =1.33–19.31) (Table 4). The frequent use of soap while bathing was not associated with reduced risk of BU (*p* = 0.69). In contrary, a significant decrease of risk of *M. ulcerans* infection was observed when using detergents for washing clothes or dishes (OR = 0.38, 95% CI = 0.32–0.45) (Table 4). Walking in stagnant water or wading in mud did not significantly increase risk of *M. ulcerans* infection (*p* = 0.72). However, frequently crossing a river (OR = 1.93, 95% CI = 1.09–3.39) or swimming (OR = 1.98, 95% CI = 1.11–3.52) in a river were associated with an increased risk of BU. Receiving cuts or scratches (OR = 1.88, 95% = 1.06–3.36) near rivers represented an additional increasing risk for contracting BU (Table 4).

**Table 4:** Univariate analysis of risk factors for Buruli ulcer disease in Zio and Yoto districts of Maritime Region, Togo, May 19–30, 2015

Characteristics	Cases n (%)	Controls n (%)	Univariable OR (95% CI)	p*
Farming	77 (92.8)	120 (93.8)	0.86 (0.29–2.56)	0.78
Farming activities				
Plowing	79 (83.1)	117 (91.4)	0.46 (0.19–1.01)	0.07
Sowing	73 (88.0)	123 (96.1)	0.29 (0.09–0.90)	**0.03**
Harvesting	74 (89.2)	125 (97.7)	0.19 (0.05–0.75)	**0.01**
Exposure to water				
Primary source of drinking water				
River or stream	38 (45.8)	58 (45.3)	1.02 (0.58–1.77)	0.95
Open borehole	15 (18.1)	23 (18.0)	1.0 (0.49–2.01)	0.98
Borehole with pump	75 (90.4)	119 (93.0)	0.71 (0.26–1.92)	0.49
Primary source of washing water				
River or stream	42 (50.6)	60 (46.9)	1.16 (0.68–2.02)	0.59
Open borehole	21 (25.3)	28 (21.9)	1.21 (0.63–2.31)	0.56
Borehole with pump	73 (88.0)	116 (90.6)	0.75 (0.31–1.83)	0.54
Bathing with a water from an open borehole	9 (10.8)	3 (2.3)	5.07 (1.33–19.31)	**0.01**
Standing water in house	10 (12.0)	18 (14.1)	0.86 (0.38–1.97)	0.72
Swam, waded or bathed in a river or stream	37 (44.6)	37 (28.9)	1.98 (1.11–3.52)	**0.02**
Crossed a body of water	48 (57.8)	55 (43.0)	1.93 (1.09–3.39)	**0.02**
Received cuts, scratches and thorn pricks near a river	49 (62.8)	60 (47.2)	1.88 (1.06–3.36)	**0.03**
Exposure to insect bite				
Received insect bite near a river	50 (64.9)	59 (46.5)	2.13 (1.19–3.83)	**0.01**
location of insect bite on the body				
Head	48 (57.8)	56 (43.8)	1.76 (1.01–3.08)	0.05
Forearms	50 (60.2)	57 (44.5)	1.88 (1.08–3.31)	**0.03**
Arms	50 (60.2)	59 (46.1)	1.77 (1.01–3.10)	**0.04**
Hands	49 (59.0)	57 (44.5)	1.79 (1.03–3.14)	**0.04**
trunk	48 (57.8)	57 (44.5)	1.70 (0.98–2.98)	0.06
thigh	48 (57.8)	56 (43.8)	1.76 (1.01–3.08)	0.05
Legs	48 (57.8)	57 (44.5)	1.70 (0.98–2.98)	0.06
Feet	48 (57.8)	57 (44.5)	1.70 (0.98–2.98)	0.06
Mosquito bite in house	75 (90.4)	124 (96.9)	0.95 (0.48–1.89)	0.90
Exposure to animals				
Owned livestock or pets	60 (77.9)	100 (78.7)	0.95 (0.48–1.89)	0.89
Handled livestock or pets	12 (15.6)	26 (20.6)	0.71 (0.33–1.50)	0.37
Share indoor living space with livestock or pets	24 (32.4)	37 (29.4)	1.15 (0.62–2.14)	0.65
Bitten or scratched by animals	5 (6.7)	10 (7.8)	0.84 (0.27–2.56)	0.76
Exposure to infectious agents				
BCG vaccination	41 (51.2)	64 (50.4)	1.03 (0.59–1.181)	0.90
Soap use while bathing				
Sometimes	5 (6.4)	10 (7.9)	1	
Always	73 (93.6)	117 (92.1)	0.80 (0.26–2.43)	0.69
Soap use while washing				
Sometimes	5 (6.0)	0 (0.0)	1	
Always	78 (94.0)	128 (100.0)	0.38 (0.32–0.45)	**0.01**
Clothing worn while farming				
Trousers	40 (48.2)	92 (71.9)	0.36 (0.20–0.65)	**0.001**
Top shirt	76 (91.6)	120 (93.8)	0.72 (0.25–2.01)	0.55
Closed shoes	9 (10.8)	26 (20.3)	0.48 (0.21–1.08)	0.07
Dress	34 (41.0)	48 (37.5)	1.16 (0.66–2.03)	0.61
Open shoes	73 (88.0)	110 (85.9)	1.19 (0.522–2.73)	0.67
Hat	6 (7.2)	35 (27.3)	0.21 (0.08–0.52)	**0.001**
Clothing worn in non-farming activity				
Trousers	33 (39.8)	61 (47.7)	0.72 (0.41–1.27)	0.26
Top shirt	73 (88.0)	118 (92.2)	0.62 (0.25–1.56)	0.31
Closed shoes	4 (4.8)	5 (3.9)	1.25 (0.32–4.78)	0.74
Dress	33 (39.8)	54 (42.2)	0.90 (0.51–1.59)	0.73
Open shoes	72 (86.7)	114 (89.1)	0.80 (0.35–1.87)	0.61
Hat	1 (1.2)	7 (5.5)	0.21 (0.25–1.75)	0.15
Insect protection products use				
Sometimes	74 (96.1)	118 (92.2)	1	0.27
Always	3 (3.9)	10 (7.8)	0.59 (0.22–1.64)	
Bednets use				
Sometimes	40 (51.3)	76 (59.4)	1	
Always	38 (48.7)	52 (40.6)	0.72 (0.41–1.27)	0.26
Perception and etiology of the BUD	68 (88.3)	113 (88.3)	1.00 (0.42–2.42)	0.99
Behavior and beliefs				
Poor hygiene cause Buruli ulcer	57 (81.4)	116 (91.3)	0.42 (0.17–0.99)	**0.04**
Seeking treatment with plants	4 (5.3)	10 (7.9)	0.64 (0.19–2.12)	0.47

*Boldface type indicates differences that were statistically significant at *p* < 0.05 between cases and controls

#### Exposure to insects

Our study showed that receiving insect bites near a river was significantly increase risk of *M. ulcerans* infection (OR = 2.13, 95% CI = 1.19–3.83) (Table 4). This risk was higher when it occurred on the forearm (OR = 1.88, 95% CI = 1.08–3.31), the arm (OR = 1.77, 95% CI = 1.01–3.10) and the hands (OR = 1.79, 95% CI = 1.03–3.14) compared to the other parts of the body. We found that mosquito bites at home were not associated with an increased risk of *M. ulcerans* infection (*p* = 0.90) (Table 4). The use of mosquito coils (*p* = 0.27) or bednets (*p* = 0.26) did not provide any significant reduction in the risk of contracting BU (Table 4).

#### Farming activities

Farming (93.8%) was the main activity of the participants of the study. However, there was no significant difference in practicing this activity between patients and controls (*p* = 0.78) (Table 4). In addition, some tasks such as sowing (OR = 0.29, 95% CI = 0.09–0.90) or harvesting (OR = 0.19, 95% CI = 0.05–0.75) during farming showed significant decrease in the risk of contracting BU (Table 4). Frequently wearing trousers (OR = 0.36, 95% CI = 0.20–0.65) or a hat (OR = 0.21, 95% CI = 0.08–0.52) while performing farming activities provided significant reduction in the risk. However, wearing clothes at home or in non-farming activities did not provided any significant reduction in the risk of BU disease (Table 4).

#### Exposure to animals

In our study, we found that living with (*p* = 0.89) or sharing indoor living space with livestock (*p* = 0.37) did not represent a significant increase in the risk of *M. ulcerans* infection neither did incurring bites or scratches from (*p* = 0.76) (Table 4). Also, hunting or handling of wild animals (*p* = 0.65) was not significantly associated with an increasing risk of BU infection.

#### BCG vaccination

Most of participants showed BCG vaccine scars and there was no significant difference between cases and controls (*p* = 0.90) (Table 4).

#### Attitude, behavior and beliefs of BUD

Of the participants interviewed, 88.3% were familiar with BU symptoms and this attitude was similar between BU cases and controls (*p* = 0.99). Regarding treatment behaviors, most of cases (83.5%) indicated seeking help from hospital while 5.3% believed in herbal treatment as the first preferred treatment option (Table 4). Considering the hygiene practice, BU cases as well as controls thought that personal poor hygiene and dirty surroundings could increase the risk of contracting BU (Table 4).

### Multivariate analysis

After adjustment for potential confounders, we found that factors such as age (< 10 years (aOR = 11.48, 95% CI = 3.72–35. 43) and 10 to 14 years (aOR = 3.63, 95% CI = 1.22–10.83)), receiving insect bites near a river in children aged 10 to14 years (aOR = 7.8, 95% CI = (1.48–41.24)) and bathing with water from open borehole (aOR = 5.77, 95% CI = 1.11–29.27) (Table 5) remain as potential factors of increasing risk of *M. ulcerans* infection.

**Table 5:** Multivariate model for risk factors of Buruli ulcer disease in Zio and Yoto Districts of the Maritime Region, Togo, May 19–30, 2015

Characteristics	aOR (95% CI)	p*
Age (Years)		
< 10	11.48 (3.72–35.43)	**0.001**
11–14	3.63 (1.22–10.83)	**0.02**
15–24	1.07 (0.39–2.97)	0.88
> 25	1	
Receiving insect bites near a river (Yes/No)		
< 10 (years)	3.29 (0.77–14.04)	0.11
**11–14 (Years)**	**7.80 (1.48–41.21)**	**0.016**
15–24 (Years)	3.05 (0.71–12.99)	0.13
> 25 (Years)	1.76 (0.48–6.45)	0.39
Bathing with water from open borehole	5.77 (1.11–29.27)	**0.03**

*Boldface type indicates differences that were statistically significant at *p* < 0.05 between cases and controls

## Discussion

The objective of this study was to identify risk factors for Buruli ulcer in the two endemic districts of Zio and Yoto of the Maritime region. This is the first study that has investigated these factors in Togo. In general, socio-demographic, behavioral or environmental factors have been considered as important risk factors for *M. ulcerans* infection.

### Socio-demographic factors

The present study showed that children under 15 years of age were at higher risk of contracting Buruli ulcer than adults. This result is in accordance with other studies conducted in Benin [11] and Ivory Coast [13] as well as WHO reports [37]. Indeed, in this age group children appeared to be often less protected especially at the head and feet [11]. Also, children’s behavior is usually driven by their parents’ activities as they accompanied them to the river for washing and for farming where they were highly exposed to aquatic areas that are associated with an increasing risk of BU infection.

### Environmental factors

We found that bathing with water from an open borehole was associated with higher risk of contracting BU. Similar results were found in Ghana [38], Ivory Coast [39] and Cameroon [14]. Indeed, other studies [6, 28, 40] have also shown that using unprotected water sources for bathing was associated with *M. ulcerans* infection. It has also been observed that even when used with soap, unprotected water sources constitute an increased risk of *M. ulcerans* infection [37]. However, Raghunathan et al. [38] in Ghana found that using a detergent while bathing provides significant reduction in Buruli ulcer risk. This difference could be explained by the antibacterial power of the soap used. Besides, in our study people from villages commonly used the local soap. On the other hand, we found that using soap to wash clothes or dishes was reducing the risk. This time, the type of the soap used for the laundry is provided from commercial brands which are strongly enriched in detergents and acids. Our study also identified other water sources of *M. ulcerans* infection such as swimming in a river, frequently crossing a river, receiving insect bites or injuries of cuts near rivers. However, after adjustment for potential confounders, only receiving insect bites near a river remained as an independent predictor of acquiring BU infection. Similar results were found in Ghana [38] but in Ivory Coast [13] and in other study [12], it was found that swimming or wading in water did significantly increase the risk of BU infection. To explore the difference of our finding with other studies, we looked to determine any potential age confounding or effect modification. Therefore, we found that insect bites increase the risk of BU only in 101-14 years age group (aOR = 7.80, 95%CI = 1.48–41.21). Though, other studies did not determine in which age group swimming or wading in water significantly increased the risk of BU, we could explain the difference between these studies by the age of BU cases. Further, in our study, 63% of BU cases were aged < 15 years while in Ivory Coast, 75% of cases were aged more than 15 years who are able to swim or wad in a river.

Most of the people surveyed were perform agricultural activities. However, we did not find any significant association with the risk of contracting BU. Among agricultural activities, planting and harvesting activities were associated with decrease risk of *M. ulcerans* infection. Similar results were found in Cameroon [14]. We observed that wearing a long-sleeved shirt or a long dress while performing agricultural activities did not provide significant reduction of the risk of contracting of Buruli ulcer. This observation is in accordance with the study conducted in Cameroon [14]. On the other hand, we found that wearing pants or hats is associated with reduction in the risk of mycobacterial infection. This would explain the low frequency of wounds on head and legs observed in our investigation. These results are consistent with those found in Ghana [12, 38] and Ivory Coast [13].

In Australia, Lavender et al. [20] showed that mosquito bites were significantly associated with Buruli ulcer. However, we did not find any risk of *M. ulcerans* infection associated to mosquito bites in Togo. In general, results of studies on mosquito bites associated with the use of mosquito coils or bednets during *M. ulcerans* infection are often contradictory [11, 14, 38, 39, 41].

Some studies [12, 13, 42] have shown that animals such as chickens, goats, cats and pigs could harbour *M. ulcerans* and exposure to these animals may increase the risk of contracting BU disease. During this study, we did not observe significant increase in risk of contracting BU associated with contact with domestic animals.

BCG vaccine is delivered against a mycobacterium. This vaccination could therefore provide a cross-protection against *M. ulcerans* infection [43]. In our study, we did not observe any significant difference in the percentage of BCG vaccination scar between patients and controls. The lack of a significant association with BCG vaccination with *M. ulcerans* infection has been also described in the literature [12, 16, 43]. However, data from Benin [11], Ivory Coast [13] and Cameroon [14] showed negative correlation between BCG vaccination and BU. Studies conducted to explore this possible cross-protection have often led to contradictory results. Indeed, a multicenter study [44] conducted in the DR Congo, Ghana and Togo did not reveal any significant association between BCG vaccination and BU disease.

### Attitude, behavior and belief on BU

The attitude of the participants interviewed has considerably improved with their capacity to recognize some BU symptoms and their ability to refer suspected cases to medical treatment compared to the situation 5 years before [33]. This finding could be attributable to several awareness campaigns in the community that had influenced their behavior toward this disease [33]. However, there remains some effort to help recognizing early symptoms by the community as well as the herbalists because 5.3% of BU patients continue to believe in herbal treatment as the first preferred treatment option. Poor individual hygiene and dirty surrounding were recognized as a potential risk factor for participants in the present study. The impact of poor hygiene and its possible role as a risk factor has been underlined in studies in Benin [11, 45] and Ghana [12].

This study had some limitation. We did not reach all participants especially some BU cases due to their unavailability during the survey time. The sample size was calculated based on the proportion of households using water from unprotected sources which was higher than the prevalence of BU. The number of newly confirmed BU cases in Togo every year is low and varies from 30 to 65 patients. During the study period, we found 91 BU cases but 8 patients were not available at the survey time. The main concern with the limit number of controls was due to the fact that in many households, there were often two to three patients and exceptionally in one house up to six. In those households, it was difficult to enroll two folds of controls. Moreover, as 47% of BU patients were under 10 years, it was difficult to interview children who were not capable to describe their activities which are driven by their parent’s duties. The reason we had decided to use their parents as controls sometimes.

## Conclusions

Our study identified some significant risk factors for BU infection including age, bathing with water from open boreholes and receiving insect bites near a river in Zio and Yoto Districts of the Maritime Region in south Togo.

## Additional file

Additional file 1: Questionnaire form S1. Questionnaire form used to collect data during the survey on risk factor for *Mycobacterium ulcerans* infection in Zio and Yoto districts of Maritime Region, Togo, May 19–30, 2017. (DOC 84 kb)

## Abbreviations

ASC: Health community volunter
BCG: Bacille calmette et guérin
CHR: Centre hospitalier régional
CNRT-UB: Centre national de référence pour le traitement de l’ulcère de buruli
DAHW-Togo: Association allemande de lutte contre la lèpre
DITM: Department of infectious and tropical medicine
EDST: Enquête de développement sanitaire du Togo
ESTBA: Ecole supérieure des techniques biologiques et alimentaires
INH: Institut national d’hygiène
OR: Odds Ratio
PCR: Polymerase chain reaction
PNLUB-LP: Programme national de lutte contre l’ulcère de buruli, la lèpre et le pian
SPSS: Statistical package for social science
VIH: Human immunodeficiency virus
WHO: World Health Organisation
