# Supplementary material for: Risk factors for Mycobacterium ulcerans infection (Buruli Ulcer) in Togo ─ a case-control study in Zio and Yoto districts of the maritime region
Source: BMC Infect Dis. 2018 Jan 19;18:48. doi: 10.1186/s12879-018-2958-3 (PMC5775556; doi:10.1186/s12879-018-2958-3)
Supplement: Additional file 1: Questionnaire form S1. — Questionnaire form used to collect data during the survey on risk factor for Mycobacterium ulcerans infection in Zio and Yoto districts of Maritime Region, Togo, May 19–30, 2017. (DOC 84 kb) [file 12879_2018_2958_MOESM1_ESM.doc]

| **Number /___/___/____/ QUESTIONNAIRE FORM** |
| --- |
| **Participant : BU Case Control** |
| **Socio-demographic informations**  Age (in years): /_________________/  Sexe : Male Female  Occupation : For children <18 years, report parent occupation (father/Mother/tutor) : Farming occupation Fishing occupation Mining occupation Hunting occupation  Marital statut: Not Married Married Widower / widow / divorced  District of residence : Give name of Village/District /Region : /_____________/_____________/_____________/  Ethnicity : /_____________________/  Educational level : No education Primary Secondary (College) Tertiary (High level)  Number of people in the household : /_____________/ |
| **Exposure to water**  Is there a stagnant water/mud in your house floor: Yes No  Primary source of drinking water: River or Stream Open borehole Borehole with pump  Primary source of washing water (laundry/dishes/kitchen): River or Stream Open borehole Borehole with pump  Do you wade or swim in?: River or Stream Stagnant water/mud  Do you bathe with water from open borehole? Yes No  Have you crossed a body of water of a river or stream? Yes No |
| **Exposure to insects/Mosquito**  Did you received cuts, scratches, thorns pricks or wounds near a river or stream: Yes No  Did you received insect bites near a river or stream: Yes No  If Yes, which part of your body was affected? Head Forearms Arms Hands Trunk Thigh Legs Feet  In which part of your body do you receive mosquito bites at home? Head Forearms Arms Hands  Trunk Thigh Legs Feet |
| **Exposure to animals**  Did you owned livestcok or pets? Yes No  Did you handled livestock or pests? Yes No  Do you Share indoor living space with livestock or pets? Yes No  Do you Share indoor living space with poultry? Yes No  Have you been Bitten or scratched by animals? Yes No |
| **Exposure to infectious agents**  Do you have a BCG scar? (Verify on the left shoulder) : Yes No    Do you know your HIV statut ? Yes No If Yes, indicate your profile : Positive Negative |
| **Protection Measures**  Do you use soap while bathing? Rarely Sometimes Always  Do you use soap for washing/laundry/dishes/kitchen? Rarely Sometimes Always  Clothing worn while farming : Trousers Top shirt Closed shoes Dress Open shoes Hat  Clothing worn while walking in the bush : Trousers Top shirt Closed shoes Dress Open shoes Hat    Clothing worn in nonfarming activities: Trousers Top shirt Closed shoes Dress Open shoes Hat |
| **Insect protection**  Do you use insect repellent or mosquito coils: Never Sometimes Always  Do you use bednets : Never Sometimes Always |
| **Behavior and beliefs**  Do you recongize Buruli ulcer symptoms? Yes No  Where do you seek treatment while recognizing BU symptoms? Hospital Herbalist Nothing    Do you beleive that poor hygiene could increase BU infection risk? Yes No  Do you  seek for or beleive on treatment with plant by herbalist? Yes No  If yes, do you know the name of plant that are used? /____________________________/  Do you know an herbalist who treat BU? Yes No |
| **Other activities**  Participating in farming activities: Digging Weeding Sowing Harvesting Plowing  Participating in mining: Yes No |
